# Distinguishing Quantitative Electroencephalogram Findings between Panic Disorder and Generalized Anxiety Disorder

**DOI:** 10.1192/j.eurpsy.2025.2118

**Published:** 2025-08-26

**Authors:** H. Suh, K.-S. Lee

**Affiliations:** 1 Psychiatry, CHA University, School of Medicine, Seoul, Korea, Republic Of

## Abstract

**Introduction:**

It is important to have early diagnosis and early intervention for generalized anxiety disorder(GAD) and panic disorder(PD). However, it is difficult to distinguish GAD from PD. Neurobehavioral markers that differentiate GAD and PD would be helpful to refine classification schemes based on neurobiological measures.

**Objectives:**

The aim of this study is to determine the distinguishing neurophysiological characteristics between generalized anxiety disorder and panic disorder using quantitative EEG.

**Methods:**

The study included 36 patients with GAD and 25 patients with PD. Resting vigilance controlled EEG recordings were assessed at 64 electrode sites according to the international 10/20 system. QEEG were compared between GAD and PD groups by frequency bands (delta 1-3 Hz, theta 4-7 Hz, alpha 8-12 Hz, beta 12-25 Hz, high beta 25-30 Hz, gamma 30-40 Hz and total 1-40 Hz) made by spectral analysis.

**Results:**

The absolute powers of theta and alpha bands at the frontal area differed between GAD and PD group. The absolute power of the theta activity was decreased in FP1 and FP2 (p < 0.05) and the absolute power of the alpha activity was decreased in F3 (p < 0.05) in cases with GAD compared to PD.

**Conclusions:**

The differences in QEEG power suggest that underlying pathophysiologic mechanisms may be different between GAD and PD. The findings that the decreased absolute powers of the theta and alpha activity at the frontal area in GAD may be the main neurophysiological characteristics of the GAD and help to have early differential diagnosis between GAD and PD.

**Disclosure of Interest:**

None Declared

